# Changes in mineral status are associated with improvements in insulin sensitivity in obese patients following l-arginine supplementation

**DOI:** 10.1007/s00394-013-0533-7

**Published:** 2013-05-25

**Authors:** Joanna Suliburska, Paweł Bogdanski, Monika Szulinska, Danuta Pupek-Musialik, Anna Jablecka

**Affiliations:** 1 Department of Human Nutrition and Hygiene, Poznan University of Life Sciences, Wojska Polskiego 31, 60-624 Poznan, Poland; 2 Department of Internal Medicine, Metabolic Disorders and Hypertension, Poznan University of Medical Sciences, Poznan, Poland; 3 Department of Clinical Pharmacology, Poznan University of Medical Sciences, Poznan, Poland

**Keywords:** Minerals, Insulin sensitivity, Obesity, l-Arginine

## Abstract

**Purpose:**

The aim of this study is to evaluate the long-term influence of l-arginine intake on mineral concentration in patients with obesity and to assess the changes in lipid serum levels, fat content, and insulin resistance that result.

**Methods:**

A randomized double-blind placebo-controlled study was conducted. 88 obese patients were randomly assigned to receive either 9 g of l-arginine or placebo daily, for 6 months. At baseline and after 6 months, selected anthropometrical measurements and blood biochemical analyses were performed and mineral levels were assessed. To assess insulin sensitivity, the gold-standard euglycemic clamp methodology was used.

**Results:**

We found that 6 months of l-arginine supplementation resulted in significant increases in insulin sensitivity (Δ1.1 mg/kg/min, *P* < 0.01) and zinc levels (Δ1.5 μmol/L, *P* < 0.001). Moreover, a positive correlation between the change in zinc concentration in serum and the change in insulin sensitivity was observed (*R* = 0.80, *P* < 0.01). In the group of patients treated with l-arginine, a negative correlation between the change in zinc concentration in serum and the change in body fat content was noted (*R* = −0.38, *P* < 0.05).

**Conclusions:**

l-Arginine supplementation affects zinc status in obese patients. One beneficial influence is related to the improvements in insulin sensitivity.

## Introduction

Obesity is a very serious problem throughout the world. It has been linked to great increases in chronic health conditions and to significantly higher health expenditures. The adverse health consequences associated with obesity include cardiovascular disease, type-2 diabetes, hypertension, dyslipidemia, cancers, and respiratory problems. Increasing evidence shows that obesity is associated with chronic low-grade inflammatory responses, oxidative stress, and insulin resistance [1, 2]. Large epidemiological studies have shown that the risk of diabetes, and presumably of insulin resistance, rises with body fat content. Insulin is a critical regulator of virtually all aspects of adipocyte biology [3].

It is known that trace minerals are involved in the pathogenesis of obesity, insulin resistance, and other metabolic disorders. The association between overweight, obesity, and deficiencies of iron, calcium, and zinc has been observed in clinical and epidemiological studies [4]. Several studies have suggested that calcium may play a role in the regulation of abdominal fat mass in obese people [5]. It has been recognized that iron influences glucose metabolism and that iron stores are associated with insulin sensitivity, insulin secretion, and type-2 diabetes [6]. Zinc and copper ions are also involved in glucometabolic disorders [7, 8]. Epidemiological studies show that magnesium intake is inversely associated with insulin resistance [9]. An association between mineral status and the high-sensitivity C-reactive protein (an inflammation marker and predictor of cardiovascular risk) has also been demonstrated [10].

Understanding these pathological processes of obesity and its co-morbidities is crucial for better prevention and treatment of patients with obesity. In several studies, it has been found that l-arginine is a substrate for nitric oxide production, a fact which has contributed to a significant growth of interest in l-arginine in the treatment and prevention of diseases of the cardiovascular system. An increasing quantity of evidence points to the potential benefits of the use of l-arginine in patients with hypertension, type-2 diabetes, atherosclerosis, and hypercholesterolemia [11, 12]. The potential use of l-arginine in patients with obesity seems in particular to be very promising. It is worth noticing that there is a lack of research assessing the potential of l-arginine supplementation in patients with obesity, and also a dearth of studies focusing on the effects of oral l-arginine supplementation on mineral concentrations in obese individuals. Considering the widespread nature of the obesity epidemic, we have carried out a study to answer the question of whether long-term l-arginine intake can influence mineral concentrations in patients with obesity and to evaluate the resulting changes in lipid plasma levels, fat content, C-reactive protein level, and insulin resistance. This article is part of a series of works on the effects of l-arginine on selected parameters in obese patients.

## Subjects and methods

### Study patients

Informed consent was obtained from all subjects, and the study was approved by the Research Ethics Committee of Poznań University of Medical Sciences (registered as no. 221/10). The study conformed to all ethical issues was included in the Helsinki declaration.

88 patients with simple obesity were studied from the outpatient clinic of the Department of Internal Medicine, Metabolic Disorders, and Hypertension at Poznań University of Medical Sciences. The inclusion criteria were as follows: (1) age 30–60 years, (2) body mass index (BMI) ≥30 kg/m^2^, (3) stable bodyweight (less than 3 kg self-reported change during the previous 3 months), (4) no need for any pharmacological treatment.

The exclusion criteria were as follows: (1) suspicion of secondary obesity (based on the patient’s history, physical examination, and available laboratory analysis), (2) arterial hypertension, (3) diabetes, (4) impaired glucose tolerance, (5) history of coronary artery disease, (6) stroke (including transient ischemic attack), (7) congestive heart failure, (8) arteriosclerosis obliterans, (9) sleep apnea, (10) abnormal liver function (abnormal alanine aminotransferase, aspartate aminotransferase, or bilirubin concentration), (11) renal failure (serum creatinine >115 μmol/L for men or >107 μmol/L for women, glomerular filtration rate <60 mL/min/1.73 m^2^), (12) thyroid gland dysfunction, (13) clinically significant inflammatory processes within the respiratory, digestive, or genitourinary tract, or else in the oral cavity, pharynx, or paranasal sinuses, (14) a history of infection within a month before the study, or history of use of any dietary supplements in the 3 months prior to the study, (15) smoking, (16) any other condition that in the opinion of the investigator would make participation not in the best interest of the subject, or could prevent, limit, or confound the protocol-specified efficacy assessments.

### Study design

A prospective, randomized double-blind design was applied. Randomization was performed by an independent statistician. Both patients and investigators were blinded to randomization.

All patients were randomized to receive 9 g l-arginine (*Curtis Healthcare, Poland*) three times daily (the study group), or else a placebo (the control group), for 6 months. The placebo consisted of pure microcrystalline cellulose. The dosage of 9 g three times daily was chosen as optimal for the patients, as it is nearly twice the amount of arginine found in average food rations in Poland. The dose appeared to be effective and safe for the patients. All treated subjects underwent a consultation with a dietician and were instructed to maintain their diet and physical activity through the study. Every 14 days, the patients were weighed to monitor their body mass throughout the study. Every 14 and 3 days before the laboratory tests, the subjects’ compliance with their dietary recommendations was determined by obtaining a 24-h dietary recall from the subjects. The amount of nutrients in the daily diet was processed and evaluated using the computer program *Dietetyk*. The intake of protein and arginine during the study was constant. The average daily protein intake was 72.5 g in women and 91.9 g in men, and the average arginine intake was 43.3 mg/kg body weight.

The use of the supplement was monitored. On the first day of the study, during the interview with each patient, a physician provided information about taking the supplement and described the benefits for the patient of using a daily l-arginine supplement. Each 14 days, the patient visited a dietician and returned the supplement packaging. The dietician recorded the amount of supplement consumed by the patient over the 2-week period. Moreover, during the first visit to the dietitian, each patient received a diary in which to enter the time they took the supplement each day. The diary was checked by the dietician or physician on the subsequent visit.

At the baseline, and after 6 months of treatment, anthropometric parameters and all laboratory tests were performed for both groups.

### Anthropometric measurements

Anthropometric measurements were conducted with individuals wearing light clothing and no shoes. Weight was measured to the nearest 0.1 kg, and height to the nearest 0.1 cm. BMI was calculated as the weight divided by the height squared (kg/m^2^). Obesity was defined as BMI ≥30 kg/m^2^. Dependent (%FAT) fat content was determined by impedance analysis, using a Bodystat analyzer (1500 *MDD; Bodystat, Isle of Man*).

### Biochemical measurements

All participants had blood collected from a forearm vein in serum-separated tubes (without using an anticoagulant). The coagulated blood was left to clot at room temperature for 30 min and then centrifuged for 15 min at 2,000 r.p.m. at 4 °C. The supernatant fluid was then separated. Blood samples were collected following an overnight fast and after 30 min in the supine position. Serum samples were stored at −20 °C for no longer than 2–3 days.

Serum levels of total cholesterol (TC) and triglyceride (TG) were assayed by routine enzymatic methods. Levels of blood glucose were determined by routine enzymatic methods.

Insulin sensitivity was evaluated by the euglycemic hyperinsulinemic clamp technique, as described elsewhere [13]. On the morning of the study, two venous catheters were inserted into the antecubital veins: the first for infusing insulin and glucose and the second in the contralateral arm for sampling blood; that arm was heated to approximately 60 °C. Insulin (Actrapid HM, Novo Nordisk, Copenhagen, Denmark) was given as a primed-continuous intravenous infusion for 3 h. Arterialized blood glucose was obtained every 5 min. The glucose infusion rate approached stable values during the final 40 min of the study, and the rate of whole-body glucose uptake (the *M* value) was calculated as the mean glucose infusion rate from 80 to 120 min. Plasma insulin was determined by immunoradiometric assay (*DIAsource immunoassays S.A.,* *Nivelles, Belgium*). Serum high-sensitivity C-reactive protein (hs-CRP) was measured by a high-sensitivity modified laser nephelometry technique (Berhing Diagnostics, GmbH, Marburg, Germany).

The levels of iron, copper, zinc, calcium, and magnesium in serum were determined by flame atomic absorption spectrometry (Zeiss AAS-3 spectrometer with deuterium background correction). In order to obtain the concentrations of the serum bioelements, the samples were diluted (v/v 1:1) as follows: for iron, zinc, and copper analyses, 0.01 % Triton X-100 (Merck) was used, while for the magnesium and calcium analysis, aqueous solutions consisting of 0.01 % Triton X-100 (Merck) and 0.05 % lanthanum chloride (Merck) were used. The content of iron, copper, zinc, calcium, and magnesium in serum samples was determined at the following wavelengths: 248.3 nm (iron), 324.8 nm (copper), 213.9 nm (zinc), 422.7 nm (calcium), and 285.2 nm (magnesium). The accuracy of the method was verified using certified reference material (HUM ASY CONTROL 2, Randox) and was 95, 99, 94, 99, and 102 % for calcium, magnesium, iron, zinc, and copper, respectively.

### Statistical analysis

The data are shown as mean ± SD. All calculations and statistics were performed with the STATISTICA 6.0 software. Statistical analysis was carried out by means of one-way repeated-measure ANOVA. Simple associations between variables were calculated as the Spearman coefficient of correlation. A *P* value of <0.05 was regarded as significant. It was calculated that a sample size of at least 30 patients in each group would yield at least an 80 % chance of detecting a treatment effect as statistically significant at the 0.05 alpha level.

## Results

The baseline characteristics of both groups are shown in Table 1. There were no statistically significant differences between the two groups prior to the study.

**Table 1:** Baseline characteristic of both groups

	l-Arginine group (*n* = 44)	Placebo group (*n* = 44)	P
Gender (male/female)	21/23	24/20	NS
Age (years)	43.1 ± 8.6	41.5 ± 9.1	NS
BMI (kg/m^2^)	36.8 ± 6.3	36.1 ± 4.9	NS
FAT (%)	37.0 ± 9.7	35.6 ± 9.8	NS
TC (mmol/L)	5.7 ± 1.2	5.5 ± 1.0	NS
TG (mmol/L)	2.2 ± 1.6	2.0 ± 0.9	NS
Glucose (mmol/L)	5.1 ± 0.7	5.0 ± 0.5	NS
Insulin (μUI/mL)	29.2 ± 13.9	28.6 ± 16.4	NS
M (mg/kg/min)	3.2 ± 1.8	3.4 ± 1.7	NS

Data are mean ± SD

BMI—body mass index, FAT—fat content, TC—total cholesterol, TG—triglycerides, M—insulin sensitivity, NS—not significant

Table 2 shows the anthropometric and biochemical parameters in l-arginine and placebo groups before and after treatment alongside with the *P* values for “treatment” and “time” factors and their interaction obtained from ANOVA analysis.

**Table 2:** Characteristic of the groups and the data at baseline and after 6 months of treatment

Parameters	Baseline		After 6 months		ANOVA		
	l-Arginine group (*n* = 44)	Placebo (*n* = 44)	l-Arginine group (*n* = 44)	Placebo (*n* = 44)	Treatment	Time	Interaction
					*P* values		
Gender (male/female)	21/23	24/20	–	–	–	–	–
Age (years)	43.1 ± 8.6	41.5 ± 9.1	–	–	–	–	–
BMI (kg/m^2^)	36.8 ± 6.3	36.1 ± 4.9	36.6 ± 6.1	35.8 ± 5.0	0.518	0.220	0.882
FAT (%)	37.0 ± 9.7	35.6 ± 9.8	35.6 ± 9.8	35.5 ± 8.6	0.665	0.435	0.528
TC (mmol/L)	5.7 ± 1.2	5.5 ± 1.0	5.6 ± 1.0	5.4 ± 0.7	0.282	0.137	0.976
TG (mmol/L)	2.2 ± 1.6	2.0 ± 0.9	2.1 ± 1.3	2.0 ± 0.7	0.509	0.229	0.438
Glucose (mmol/L)	5.1 ± 0.7	5.0 ± 0.5	4.8 ± 0.6	4.9 ± 0.5	0.998	**0.018**	0.138
Insulin (μUI/Ml)	29.2 ± 13.9	28.6 ± 16.4	25.5 ± 12.7	26.8 ± 14.7	0.903	**0.048**	0.228
M (mg/kg/min)	3.2 ± 1.8	3.4 ± 1.7	4.3 ± 2.1	3.0 ± 1.6	**0.043**	**0.008**	**0.001**
hs-CRP (mg/l)	2.8 ± 1.2	2.9 ± 1.0	2.6 ± 1.4	2.8 ± 1.0	0.516	0.055	0.418
Ca (mmol/L)	2.4 ± 0.4	2.7 ± 0.4	2.6 ± 0.3	2.6 ± 0.3	0.140	0.066	0.088
Mg (mmol/L)	0.8 ± 0.1	0.9 ± 0.2	0.9 ± 0.1	0.9 ± 0.1	0.293	0.069	0.078
Fe (mmol/L)	14.9 ± 4.0	15.3 ± 3.2	17.7 ± 5.3	16.7 ± 3.5	0.139	0.056	0.055
Zn (mmol/L)	10.7 ± 1.1	10.5 ± 1.2	12.2 ± 1.6	10.7 ± 1.4	**0.007**	**0.000**	**0.000**
Cu (mmol/L)	14.0 ± 2.3	13.9 ± 2.6	13.0 ± 1.7	14.1 ± 2.1	0.111	**0.049**	**0.003**

Bold values indicate one-way repeated-measure ANOVA, significant differences at *P* < 0.05

Data are arithmetic mean ± SD

BMI—body mass index, FAT—fat content, TC—total cholesterol, TG—triglycerides, M—insulin sensitivity value, hs-CRP—high sensitive C-reactive protein

Statistically significant interaction between treatment and time factors (with both factors statistically significant) was observed for insulin sensitivity (*P* = 0.001) and zinc concentration (*P* < 0.001): The increase in insulin sensitivity and zinc level was significantly higher in l-arginine group compared to placebo group. Statistically significant interaction between treatment and time factors (with time factor statistically significant) was observed for copper level (*P* < 0.05): The decrease in copper concentration was observed in l-arginine group. A decrease in glucose and insulin serum concentration was observed in both l-arginine and placebo group during the course of the study with time factor statistically significant; however, no significant interaction between treatment and time factors was found (Table 2). Increased levels of iron in serum in both groups were observed, but the differences were not significant (Table 2).

Several correlations were found between minerals and other parameters in the subjects following treatment. Changes in the level of zinc in serum were markedly associated with changes in insulin sensitivity (*R* = 0.80, *P* < 0.01) and with the content of fat in the body (*R* = −0.38, *P* < 0.05). A positive correlation between zinc concentration and insulin sensitivity was observed. The concentration of zinc in serum was inversely correlated with BMI index and fat content. Higher levels of magnesium in serum were associated with lower percentages of fat in the body following treatment. A positive correlation between copper levels and TG levels in serum was also found (Table 3).

**Table 3:** Correlation between minerals and other parameters after treatment

Mineral/parameter	*R*	*P*
Zn/BMI	−0.51	<0.05
Zn/%FAT	−0.49	<0.05
Zn/M	0.62	<0.01
Mg/%FAT	−0.38	<0.05
Cu/TG	0.47	<0.05
ΔZn/Δ%FAT	−0.38	<0.05
ΔZn/ΔM	0.80	<0.01

R—correlation coefficient, Δ—change of parameter during treatment, BMI—body mass index, FAT—fat content, TG—triglycerides, M—insulin sensitivity

## Discussion

In this study, we provide evidence that 6 months of treatment with l-arginine increases insulin sensitivity in obese patients. We have demonstrated this for the first time using euglycemic clamp technique. The influence of l-arginine supplementation on increased insulin sensitivity has also been observed in other clinical and experimental studies [14–16]. In our previous study, we also observed the beneficial impact of l-arginine supplementation on insulin resistance in patients with visceral obesity [17]. In other studies, it has been shown that l-arginine stimulates insulin secretion and influences nitric oxide [18, 19].

The molecular mechanism involved in the influence of NO on insulin sensitivity has been studied intensely. In experimental studies, reduction in NO synthesis prevents glucose transportation in skeletal muscle cells [20]. de Castro Barbosa et al. [15] indicated that arginine improves glucose metabolism in skeletal muscle via the NO/c-GMP cascade. Additionally, Monti et al. [21] demonstrated that l-arginine may increase the activity of glucokinase in isolated liver cells of rats. In our previous study, we found that l-arginine supplementation improves insulin sensitivity in visceral obese patients independently of TNF-α activity [22].

In this study, we found that 6 months of treatment with l-arginine significantly increased zinc and decreased copper level in serum, while the changes in iron were slight.

In our opinion, changes in the concentration of minerals under the influence of l-arginine in obese subjects can be linked to the impact of arginine on insulin secretion and the synthesis of nitric oxide. In obese subjects, high levels of glucose and insulin were observed before treatment. At the same time, the reported concentrations of zinc and magnesium were at the lower limit of normal in serum. A recent report described reduced levels of zinc in obese, insulin-resistant subjects [23]. In type-2 diabetics, lower zinc plasma concentrations associated with increased excretion of zinc due to polyuria have been observed [8]. In the present work, zinc content in the urine was not studied, and so it is not known if increases in zinc concentration in serum were associated with a lower level of excretion of zinc from the body. However, it may be supposed that the increase of zinc in the serum of subjects was caused by changes in insulin sensitivity following arginine supplementation. In this study, zinc concentration in the serum was positively correlated with insulin sensitivity in patients following treatment with l-arginine. The relationship between zinc content and insulin sensitivity has also been observed in other studies [24, 25]. It has been found that poor zinc status is associated with an increased risk of insulin resistance and that a higher content of zinc in the organism can improve insulin sensitivity [26–28]. Other authors have determined that severe postnatal Zn deficiency can induce hyperglycemia, and mild Zn deficiency may alter glucose metabolism. Other data demonstrate that mild maternal Zn deficiencies are associated with elevated leptin concentrations and insulin resistance in male offspring in adulthood [29].

Zinc is an essential trace mineral, which is important for maintaining the structure and stability of both insulin and insulin receptors, and is directly involved in the physiology and action of insulin [29]. Insulin is stored in the form of insulin zinc crystals in the *β*-cells of the pancreas. The effect of zinc on the inhibition of glycogen synthase kinase 3β has been shown in laboratory studies. In a trial on diabetic patients, zinc supplementation has been shown to be effective in reducing HbA_1_C [30]. Interestingly, in a study conducted on obese children, supplementation with zinc caused changes similar to those observed in patients who had undergone l-arginine supplementation in the present study. In the study of obese children, zinc led to decreases in glucose, insulin, and HOMA values in the children, while BMI, waist circumference, LDL-cholesterol levels and triglyceride levels did not change significantly [30]. The mechanism of the effects of zinc and arginine on insulin resistance seems to differ, but they may be connected. We think that the underlying mechanism of the zinc/insulin sensitivity following arginine supplementation needs to be explored through broader physiological tests.

Interestingly, it has been reported that diabetics have elevated levels of copper [8]. Evidence of the close relationship between copper and insulin resistance is also provided by a study in which treatment with a copper-chelating agent, tetrathiomolybdate, decreased serum copper ion, glucose, insulin concentrations, and also triglyceride levels. The authors of the study suggest that such a copper-chelating agent may serve as a novel therapy for type-2 diabetes [7]. In our current study, we observed decreased copper concentration accompanied by increased insulin sensitivity following l-arginine supplementation. It can be assumed that this change in copper concentration might be caused by the influence of l-arginine on insulin status.

In obese patients, low levels of magnesium and zinc in the body are often observed [31, 32]. It is also known that fat tissue and its metabolism are connected with insulin resistance [33]. In this study, supplementation with l-arginine influenced insulin sensitivity and slightly decreased fat content. Following the treatment, zinc and magnesium concentrations were found to relate inversely with fat content in the body. It seems that decreased body fat is connected with increased insulin sensitivity and that this is associated with changes in zinc and magnesium levels.

In this study, we also analyzed hs-CRP levels, as obesity is often associated with elevated levels of this marker of subclinical inflammation and predictor of cardiovascular risk [34]. It was observed that the decrease in this parameter following l-arginine treatment was not significant. We also did not find a marked correlation between CRP and insulin resistance or minerals in patients with l-arginine.

Our study has some limitations: the study group should be larger, so that it could be divided by gender to allow observation of the changes that occur in women and men. We did not analyze the parameters of mineral metabolism, which would allow a broader conclusion about the influence of l-arginine on mineral status and would help to explain this mechanism.

In conclusion, l-arginine supplementation affects zinc status in simple obesity. Mineral changes are associated with insulin sensitivity improvements following l-arginine supplementation in obese patients. Further investigation is needed to explain the mechanism of influence of l-arginine on the mineral status associated with insulin improvement.

## References

[1] Gupta D, Krueger CB, Lastra G (2012). Over-nutrition, obesity and insulin resistance in the development of β cell dysfunction. Curr Diabetes Rev.

[2] Abdullah A, Wolfe R, Stoelwinder J, de Courten M, Stevenson C, Walls HL, Peeters A (2011). The number of years lived with obesity and the risk of all cause and cause specific mortality. Int J Epidemiol.

[3] Kahn BB, Flier JS (2000). Obesity and insulin resistance. J Clin Invest.

[4] de Luis DA, Pacheco D, Izaola O, Terroba MC, Cuellar L, Cabezas G (2011). Micronutrient status in morbidly obese women before bariatric surgery. Surg Obes Relat Dis.

[5] Rosenblum JL, Castro VM, Moore CE, Kaplan LM (2012). Calcium and vitamin D supplementation is associated with decreased abdominal visceral adipose tissue in overweight and obese adults. Am J Clin Nutr.

[6] Fernandez-Real JM, Lopez-Bermejo A, Ricart W (2002). Cross-talk between iron metabolism and diabetes. Diabetes.

[7] Tanaka A, Kaneto H, Miyatsuka T, Yamamoto K, Yoshiuchi K, Yamasaki Y, Shimomura I, Matsuoka T, Matsuhisa M (2009). Role of copper ion in the pathogenesis of type 2 diabetes. Endocr J.

[8] Wiernsperger N, Rapin JR (2010). Trace elements in glucometabolic disorders: an update. Diabetol Metab Syndr.

[9] Kim DJ, Xun P, Liu K, Loria C, Yokota K, Jacobs DR, He K (2010). Magnesium intake in relation to systemic inflammation, insulin resistance, and the incidence of diabetes. Diabetes Care.

[10] Zeba AN, Delisle HF, Rossier C, Renier G (2012). Association of high-sensitivity C-reactive protein with cardiometabolic risk factors and micronutrient deficiencies in adults of Ouagadougou, Burkina Faso. Br J Nutr.

[11] Böger RL (2008). Arginine therapy in cardiovascular pathologies: beneficial or dangerous?. Curr Opin Clin Nutr Metab Care.

[12] Cable D, Celotto A, Evora P, Schaff H (2009). Asymmetric dimethylarginine endogenous inhibition of nitric oxide synthase causes differential vasculature effects. Med Sci Monit.

[13] DeFronzo RA, Tobin JD, Andres R (1979). Glucose clamp technique: a method for quantifying insulin secretion and resistance. Am J Physiol.

[14] Piatti PM, Monti LD, Valsecchi G, Magni F, Setola E, Marchesi F, Galli-Kienle M, Pozza G, Alberti KG (2001). Long-term oral l-arginine administration improves peripheral and hepatic insulin sensitivity in type 2 diabetic patients. Diabetes Care.

[15] de Castro Barbosa T, Jiang LQ, Zierath JR, Nunes MT (2013). l-Arginine enhances glucose and lipid metabolism in rat L6 myotubes via the NO/c-GMP pathway. Metabolism.

[16] Monti LD, Casiraghi MC, Setola E, Galluccio E, Pagani MA, Quaglia L, Bosi E, Piatti P (2013). l-Arginine enriched biscuits improve endothelial function and glucose metabolism: a pilot study in healthy subjects and a cross-over study in subjects with impaired glucose tolerance and metabolic syndrome. Metabolism.

[17] Bogdanski P, Szulinska M, Suliburska J, Pupek-Musialik D, Jablecka A, Witmanowski H (2012). Supplementation with l-arginine favourably influences plasminogen activator inhibitor type 1 concentration in obese patients—a randomized, double blind trial. J Endocrinol Invest.

[18] Krause MS, McClenaghan NH, Flatt PR, de Bittencourt PI, Murphy C, Newsholme P (2011). l-arginine is essential for pancreatic β-cell functional integrity, metabolism and defense from inflammatory challenge. J Endocrinol.

[19] Thams P, Capito K (1999). l-Arginine stimulation of glucose-induced insulin secretion through membrane depolarization and independent of nitric oxide. Eur J Endocrinol.

[20] Balon TW, Nadler JL (1997). Evidence that nitric oxide increases glucose transport in skeletal muscle. J Appl Physiol.

[21] Monti LD, Valsecchi G, Costa S (2000). Effects of endothelin 1 and nitric oxide on glucokinase activity in isolated rat hepatocytes. Metabolism.

[22] Bogdanski P, Suliburska J, Grabanska K, Musialik K, Cieslewicz A, Skoluda A, Jablecka A (2012). Effect of 3-month l-arginine supplementation on insulin resistance and tumor necrosis factor activity in patients with visceral obesity. Eur Rev Med Pharmacol Sci.

[23] Suliburska J, Bogdanski P, Pupek-Musialik D, Krejpcio Z (2011). Dietary intake and serum and hair concentrations of minerals and their relationship with serum lipids and glucose levels in hypertensive and obese patients with insulin resistance. Biol Trace Elem Res.

[24] Padmavathi IJN, Kishore YD, Venu L, Ganeshan M, Harishankar N, Giridharan NV, Raghunath M (2009). Prenatal and perinatal zinc restriction: effects on body composition, glucose tolerance and insulin response in rat offspring. Exp Physiol.

[25] Basaki M, Saeb M, Nazifi S, Shamsaei HA (2012). Zinc, copper, iron, and chromium concentrations in young patients with type 2 diabetes mellitus. Biol Trace Elem Res.

[26] Ortega RM, Rodríguez-Rodríguez E, Aparicio A, Jiménez AI, López-Sobaler AM, González-Rodríguez LG, Andrés P (2012). Poor zinc status is associated with increased risk of insulin resistance in Spanish children. Br J Nutr.

[27] Hwang I, Yoon T, Kim C, Cho B, Lee S, Song MK (2011). Different roles of zinc plus arachidonic acid on insulin sensitivity between high fructose- and high fat-fed rats. Life Sci.

[28] Kelishadi R, Hashemipour M, Adeli K, Tavakoli N, Movahedian-Attar A, Shapouri J, Poursafa P, Rouzbahani A (2010). Effect of zinc supplementation on markers of insulin resistance, oxidative stress, and inflammation among prepubescent children with metabolic syndrome. Metab Syndr Relat Disord.

[29] Jou MY, Philipps AF, Lönnerdal B (2010). Maternal zinc deficiency in rats affects growth and glucose metabolism in the offspring by inducing insulin resistance postnatally. J Nutr.

[30] Hashemipour M, Kelishadi R, Shapouri J, Sarrafzadegan N, Amini N, Tavakoli N, Movahedian-Attar A, Mirmoghtadaee P, Poursafa P (2009). Effect of zinc supplementation on insulin resistance and components of the metabolic syndrome in prepubertal obese children. Hormones.

[31] Farhanghi MA, Mahboob S, Ostadrahimi A (2009). Obesity induced magnesium deficiency can be treated by vitamin D supplementation. J Pak Med Assoc.

[32] Ennes Dourado Ferro F, de Sousa Lima VB, Mello Soares NR, Franciscato Cozzolino SM, do Nascimento Marreiro D (2011). Biomarkers of metabolic syndrome and its relationship with the zinc nutritional status in obese women. Nutr Hosp.

[33] Hardy OT, Czech MP, Corvera S (2012). What causes the insulin resistance underlying obesity?. Curr Opin Endocrinol Diabetes Obes.

[34] Choi J, Joseph L, Pilote L (2013). Obesity and C-reactive protein in various populations: a systematic review and meta-analysis. Obes Rev.

